# Googling for Ticks and Borreliosis in Germany: Nationwide Google Search Analysis From 2015 to 2018

**DOI:** 10.2196/18581

**Published:** 2020-10-16

**Authors:** Cora Scheerer, Melvin Rüth, Linda Tizek, Martin Köberle, Tilo Biedermann, Alexander Zink

**Affiliations:** 1 Department of Dermatology and Allergy Technical University of Munich Munich Germany

**Keywords:** Google, infodemiology, infoveillance, public health, seasonal health trend, medical internet research, tick-borne disease, tick bites, borreliosis, Lyme disease

## Abstract

**Background:**

Borreliosis is the most frequently transmitted tick-borne disease in Europe. It is difficult to estimate the incidence of tick bites and associated diseases in the German population due to the lack of an obligation to register across all 16 federal states of Germany.

**Objective:**

The aim of this study is to show that Google data can be used to generate general trends of infectious diseases on the basis of borreliosis and tick bites. In addition, the possibility of using Google AdWord data to estimate incidences of infectious diseases, where there is inconsistency in the obligation to notify authorities, is investigated with the perspective to facilitate public health studies.

**Methods:**

Google AdWords Keyword Planner was used to identify search terms related to ticks and borreliosis in Germany from January 2015 to December 2018. The search volume data from the identified search terms was assessed using Excel version 15.23. In addition, SPSS version 24.0 was used to calculate the correlation between search volumes, registered cases, and temperature.

**Results:**

A total of 1999 tick-related and 542 borreliosis-related search terms were identified, with a total of 209,679,640 Google searches in all 16 German federal states in the period under review. The analysis showed a high correlation between temperature and borreliosis (*r*=0.88), and temperature and tick bite (*r*=0.83), and a very high correlation between borreliosis and tick bite (*r*=0.94). Furthermore, a high to very high correlation between Google searches and registered cases in each federal state was observed (Brandenburg *r*=0.80, Mecklenburg-West Pomerania *r*= 0.77, Saxony *r*= 0.74, and Saxony-Anhalt *r*=0.90; all *P*<.001).

**Conclusions:**

Our study provides insight into annual trends concerning interest in ticks and borreliosis that are relevant to the German population exemplary in the data of a large internet search engine. Public health studies collecting incidence data may benefit from the results indicating a significant correlation between internet search data and incidences of infectious diseases.

## Introduction

Borreliosis is the most frequently transmitted tick-borne disease in Europe. In 80%-90% of all cases, the disease presents with visible skin manifestations [1-3]. However, there is limited accurate data for the incidence of tick bites and borreliosis as associated diseases in Germany. Tick bites and associated diseases are important public health concerns because of their high incidence with no clear increasing or decreasing trend in Germany with regional variation [4]. Tick bite protection, correct and prompt tick removal, and medical consultation should be promoted by physicians and health authorities to facilitate early diagnosis and treatment of associated diseases.

In 9 out of 16 federal states in Germany (Bavaria, Berlin, Brandenburg, Mecklenburg-West Pomerania, Rhineland-Palatinate, Saarland, Saxony, Saxony-Anhalt, and Thuringia), it is mandatory to report diagnosed borreliosis to the German federal government agency and research institute for disease control and prevention (Robert-Koch Institute). Thus, epidemiologic data for tick bites and associated diseases are based on measured, as well as estimated, values.

Google search analysis is a powerful tool to reflect the German population’s interest in specific topics because of its 94% market share [5]. Additionally, the general public favors search engines like *Google* over specialized websites when searching for primary health information online [6-9]. Previous studies have already demonstrated that analysis of internet search volume, which is one of the methods of the fields of infodemiology and infoveillance, is a valid method for assessing medical topics [10-15]. The internet’s emerging role as a main, or at least primary, source of health advice for the general public has prompted a corresponding increase in its value in the medical field. Huang et al [16], for example, reported a minor association between online cancer-related information searches and skin cancer incidence. Additionally, Wehner et al [17] established that internet search volume positively correlates with the incidence and mortality rates of common cancers in the United States. Regarding infectious diseases, Ginsberg et al [18] showed an accurate estimation of weekly influenza activity in the United States correlated with queries in online search engines. They suggested that internet research could help physicians diagnose influenza earlier to prevent epidemics [18].

Ticks are only active when ambient air temperature is 4-10 °C, so average temperature should be an important factor influencing tick-related queries. Therefore, weather data should provide insight into seasonal patterns [19]. Previous studies have shown that there are seasonal patterns in Google search volumes but have not found significant correlation between mean monthly temperature and internet searches for “tick” [20].

This study aims to investigate the interest of the German population in tick bites and borreliosis by analyzing Google searches. Furthermore, this study aims to explore correlations between searches and whether that could provide information about real life tick bite occurrences, as well as associated diseases.

## Methods

### Study Design

In this retrospective study, Google AdWords Keyword Planner was used to measure the search volume of terms related to tick bites and borreliosis across Germany from January 2015 to December 2018. The Keyword Planner is often used by advertisers to improve Google marketing campaigns and provides monthly search volumes estimated by Google. The term *search volume* applies to the number of searches for a topic or search term. To assess search volume within a specific field, words are initially entered into the Keyword Planner; thereupon, the program provides keywords that are most relevant to the topic. This process may be used both to answer scientific questions and for medical research [10,11].

In addition, search terms related to tick bites were identified using a keyword cluster for the German words for “tick bite” (“Zeckenbiss”) and “borreliosis” (“Borreliose”). Based on this cluster, Google AdWords Keyword Planner determined search terms to be analyzed. This data included only Google users with a German internet protocol address who used the German language. Furthermore, the German Climate Data Centre [21] was used to relate Google search volume to weather data by analyzing mean monthly temperature in degrees Celsius. Due to seasonal differences in tick activity as well as tick bite incidence, we defined summer months as April to September and winter months as October to March.

In 9 of 16 federal states in Germany (Bavaria, Berlin, Brandenburg, Mecklenburg-West Pomerania, Rhineland-Palatinate, Saarland, Saxony, Saxony-Anhalt, and Thuringia) covering 42% of the total German population, mandatory notification for the three most common Lyme borreliosis manifestations (erythema migrans, acute neuroborreliosis, and Lyme arthritis) has been achieved since 2013.

To assess whether the Google search volume correlates with registered cases of borreliosis, all registered cases from the federal states of Brandenburg, Mecklenburg-West Pomerania, Saxony, and Saxony-Anhalt were considered in the analysis, as complete statistics of registered data were only available for these on the website of the German federal government agency and research institute for disease control and prevention (Robert-Koch Institut).

### Statistical Analysis

The search volume data of the identified search terms was assessed using Excel version 15.23 (Microsoft Corporation). To describe the relationship between the investigated variables, we used SPSS version 24.0 (IBM Corp) to calculate the Pearson correlation coefficient (*r*) [22].

## Results

In total, Google AdWords Keyword Planner identified 1999 search terms related to tick bites with a search volume of 26,080,530 in Germany from January 2015 to December 2018. The most frequently searched terms were “tick sting” (“Zeckenstich”; n=2,821,800, 10.82%), “tick” (“Zecke”; n=2,387,500, 9.15%), and “tick bite” (“Zeckenbiss”; n=178,850, 0.69%; Textbox 1).

**Top five key terms for tick bite (Zeckenbiss) and borreliosis (Borreliose).**

**Tick bite (Zeckenbiss; German translation in parenthesis)**

- “tick-bite” (“Zeckenbiss”)
- “tick sting” (“Zeckenstich”)
- “tick” (“Zecke”)
- “borreliosis” (“Borreliose”)
- “borreliosis symptoms” (“Borreliose Symptome”)

**Borreliosis (Borreliose; German translation in parenthesis)**

- “borreliosis” (“Borreliose”)
- “borreliosis symptoms” (“Borreliose Symptome”)
- “tick- bite” (“Zeckenbiss”)
- “tick” (“Zecke”)
- “symptoms borreliosis” (“Symptome Borreliose”)

**Figure 1:**
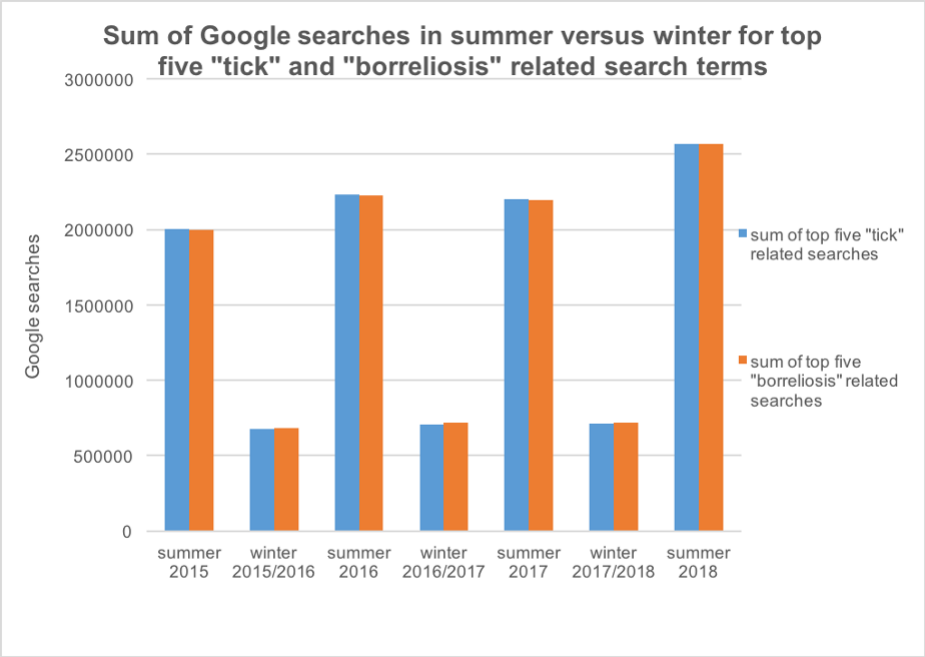
Google searches in Germany in summer (April to September) vs winter months (October to March) for the top five tick- and borreliosis-related search terms in 2015-2018.

**Figure 2:**
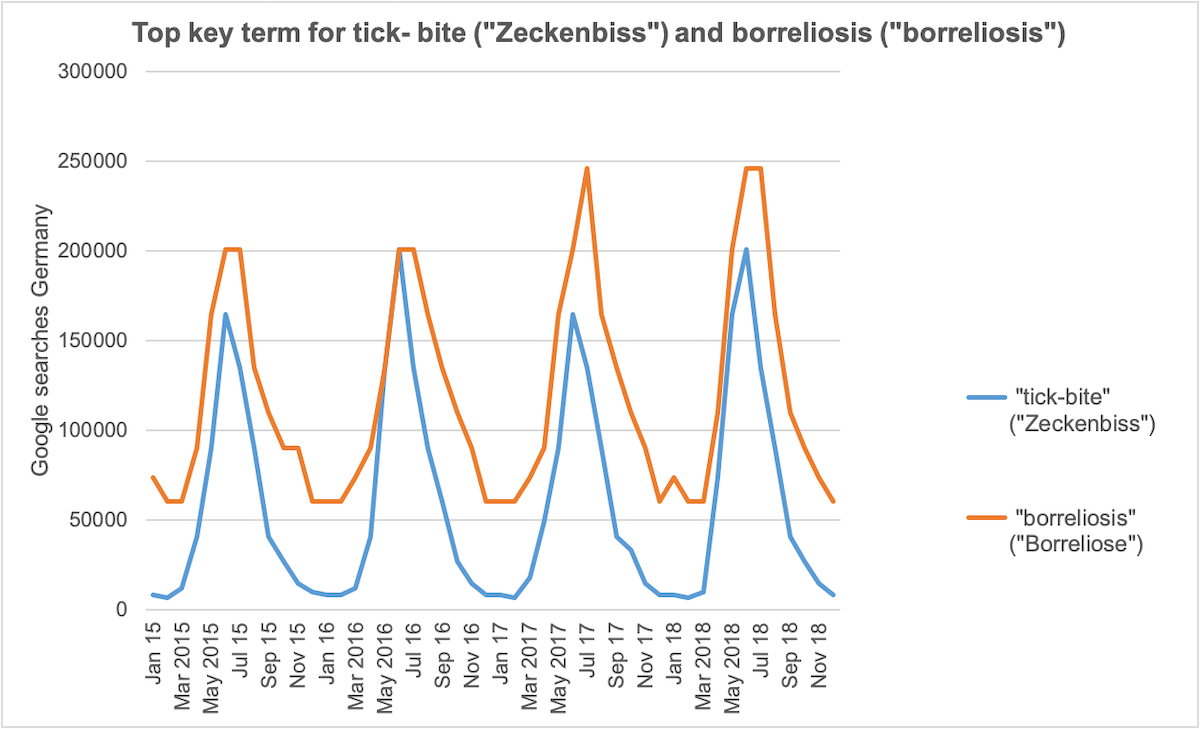
Seasonal variation of the two most common keywords searched for in Germany, “tick-bite” and “borreliosis,” from January 2015 to December 2018.

Every year, an increase in search volume during summer months was observed (Figure 1). The month with the highest overall search volume was June 2018 with 1,571,330 searches. The search volume of tick bite and borreliosis showed similar trends in search volume, with “borreliosis” being more frequently searched. Annual peaks of search volume for tick bite were seen every year in June. Annual peaks of search volume for borreliosis happened in June and July of 2015 and 2016 (n=201,000 searches each), July 2017 (n=246,000 searches), and June and July 2018 (n=246,000 searches each; Figure 2).

**Figure 3:**
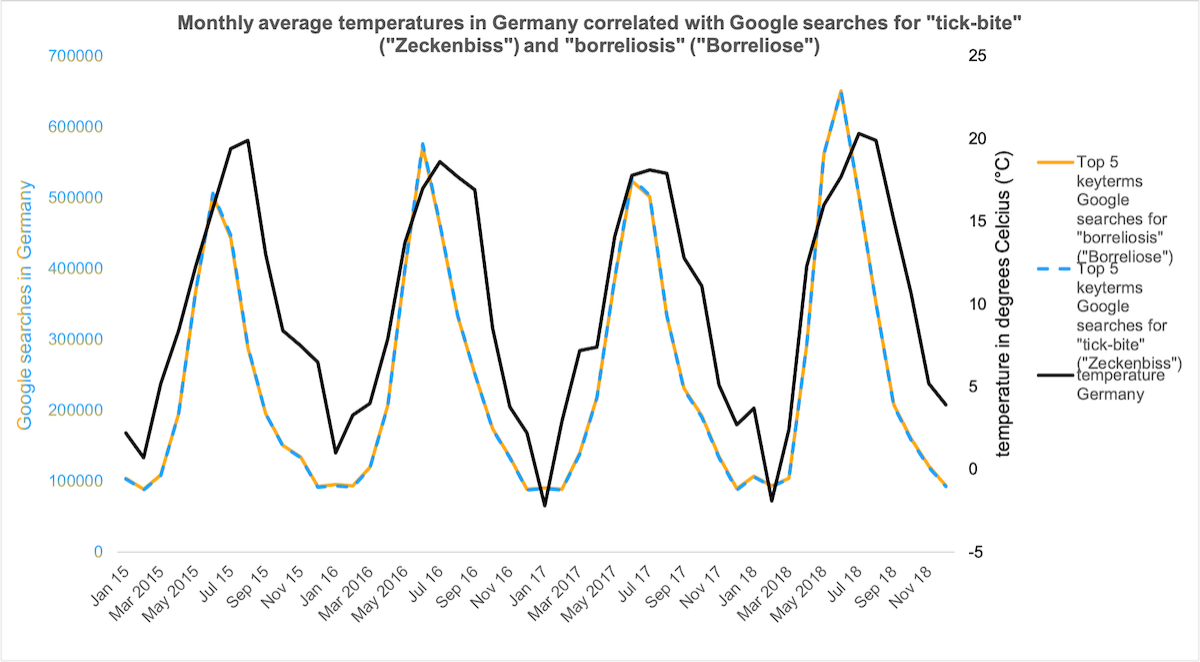
Google searches in Germany for "tick bite" and "borreliosis" correlated with the monthly average temperature in Germany in Celsius degrees between January 2015 to December 2018.

The analysis revealed a high correlation between temperature and borreliosis (*r*=0.88, *P*<.001) as well as between temperature and tick bite (*r*=0.83, *P*<.001; Figure 3). The very high correlation between borreliosis and tick bite (*r*=0.94, *P*<.001) depicts the seasonal- and temperature-dependent interest in the key terms.

**Figure 4:**
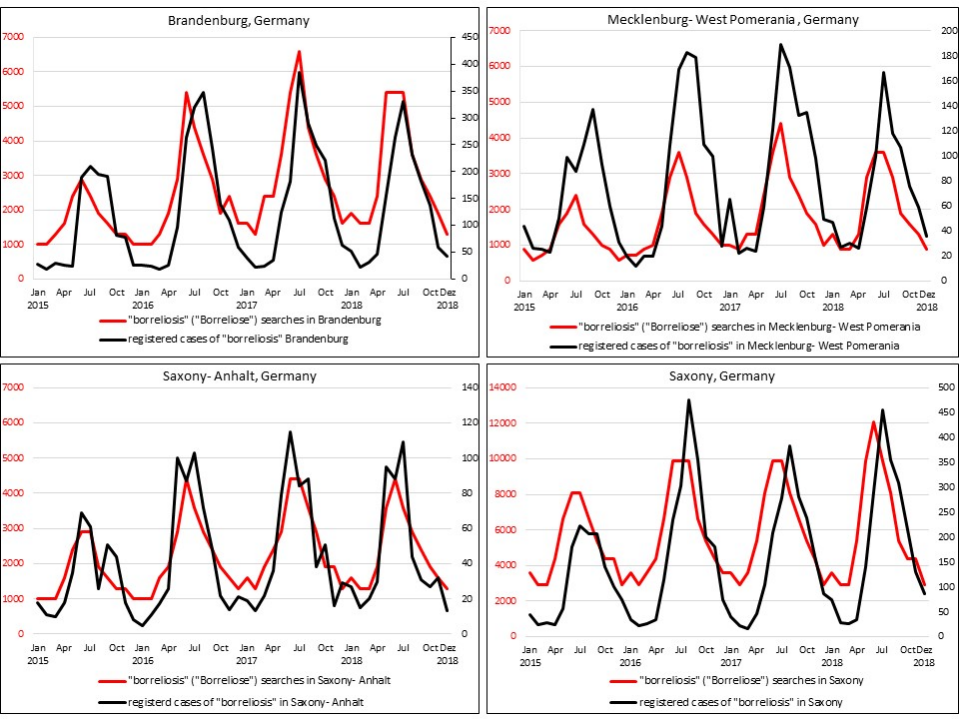
Number of Google searches for "borreliosis" with registered cases of borreliosis in Brandenburg, Mecklenburg-West Pomerania, Saxony, and Saxony-Anhalt between January 2015 to December 2018.

Furthermore, a high and very high correlation between google searches and registered cases in the referred federal states Brandenburg, Mecklenburg-West Pomerania, Saxony, and Saxony-Anhalt were detected (Figure 4; Table 1). Saxony, the federal state with the largest population, had the highest number of Google searches (n=273,800 searches), as well as the highest number of registered cases of borreliosis (n=7387). Accordingly, a high correlation was found (*r*=0.74, *P*<.001). However, the highest correlation was found in Saxony-Anhalt (*r*=0.90, *P*<.001; Table 1 and Figure 4).

**Table 1:** Pearson correlation coefficients for Google searches for borreliosis and registered cases of borreliosis from January 2015 to December 2018 in Brandenburg, Mecklenburg-West Pomerania, Saxony, and Saxony-Anhalt.

Federal state	Registered cases, n	Google searches, n	Pearson correlation	*P* value
Brandenburg	6107	124,000	0.80	<.001
Mecklenburg-West Pomerania	3720	82,480	0.77	<.001
Saxony	7387	273,800	0.74	<.001
Saxony-Anhalt	2016	104,700	0.90	<.001

## Discussion

### Principal Findings

The analysis of Google search volume related to tick bite and borreliosis identified an annual pattern that people tended to search more frequently during summer months. Therefore, a high correlation with average temperature was observed. Furthermore, a high correlation between registered cases of borreliosis in four German federal states was revealed.

One of the top five key terms of tick bite was borreliosis and vice versa. Therefore, we compared the two most common lay terms, tick bite and borreliosis. Interestingly, the search volume for the latter was higher. Especially in 2017 and 2018, a greater divergence between the keywords was observed, which can be explained by a greater awareness of associated diseases. This might be because of celebrities diagnosed with Lyme disease, such as Bastian Schweinsteiger and Justin Bieber, or because health education programs have taught people to make more accurate searches. Furthermore, media like smartphone apps and video games significantly improve knowledge of the disease and preventive measures [23,24]. However, a Finnish survey showed that, regarding knowledge, attitudes, and practice toward ticks and tick-borne disease, 65% of participants relied on newspapers and magazines as the main source of information [25]. Pharmacy health magazines, radio, or TV shows start media coverage of ticks and tick-borne diseases in late spring when average temperatures rise and ticks begin to appear. However, we did not find data to support this well-known approach.

Correlating weather data with the Google search volume showed seasonal trends, which were described in previous works concerning pruritus and identified inhabitants’ needs [12]. Especially during the German winter months October 2016 to March 2017 and October 2017 to March 2018, the Google search volume showed a distinctive increase for tick bite and borreliosis compared to data from October 2015 to March 2016. The first hypothesis was that winters get milder in Germany due to climate change so that ticks have a longer active period. Ticks are active when average temperatures are between 4-10 °C (median 7 °C) [19]. However, as during each winter, there were a comparable number of months below this temperature. This does not explain the recognizable increase in search volume that we can see. Potentially, the increase was due to the awareness of tick bites and associated diseases, as well as media campaigns starting earlier in those years.

In some German federal states, it is mandatory to report borreliosis cases. Comparing numbers from Google searches and registered borreliosis cases shows a discrepancy. For example, in Brandenburg, the highest number of registered borreliosis cases in the reviewed years was 1743 in 2017. In comparison, the 2017 Google search volume for “borreliosis” in Brandenburg was 38,200, which is 22 times higher. This might be because the number of tick bites are much greater than the development of borreliosis symptoms. Additionally, not only affected people but also their relatives might search for information online, which explains a considerably higher number of search queries.

Walker [20] posed the question of whether Google trends can be used to study parasitic (ie, tick-borne) diseases [20]. Their results showed seasonal patterns in search volume but no significant correlation between mean monthly temperature and internet searches for “tick.” Additionally, they tried to use the internet search volume to estimate parasitic occurrence. However, there was no apparent relationship between the annual number of tick-borne encephalitis cases and mean annual internet searches for either tick or tick-borne encephalitis [20].

We found statistically high correlations between registered borreliosis cases and Google search volume in four federal states. Previous studies identified Google data as a predictor of infectious disease outbreaks [26,27]. Nevertheless, to the best of our knowledge, this is the first work that shows a high correlation between incidence of an infectious disease and Google search volume of the implied disease. These results could help estimate incidences of borreliosis in German federal states where registration is not mandatory. Furthermore, Google search volume could be used to estimate incidences of diseases that are not required to be reported.

### Limitations

This study has some limitations. In Germany, Google accounts for 95% of search engine use, so Google data can depict the interests of the population as a whole. To transfer our findings to other countries, different market shares of Google over alternative search engines need to be taken into account. Although it is common among the whole population to make health-related searches, younger people tend to use the internet more often [28]. Furthermore, the automatic completion of search terms by Google may influence people’s search behavior. It may promote an understanding of the health problem and the need to seek necessary medical help; however, priming by autocomplete has the potential to make incorrect associations [29]. Another limitation to our study is that we solely used German key terms.

### Conclusion

Our study provides insight into terms and fields of interest associated with tick bites and borreliosis, relevant to the German population. We found statistically high correlations between Google searches for borreliosis and registered cases of borreliosis across four German federal states. Accordingly, these results could help to estimate the incidence of borreliosis in the remaining 12 German federal states where it is not mandatory to report borreliosis. Furthermore, this approach could aid in the development and implementation of effective and sustainable awareness campaigns.
